# Increases in IgE, Eosinophils, and Mast Cells Can be Used in Diagnosis and to Predict Relapse of IgG4-Related Disease

**DOI:** 10.1016/j.cgh.2017.02.007

**Published:** 2017-09

**Authors:** Emma L. Culver, Ross Sadler, Adrian C. Bateman, Mateusz Makuch, Tamsin Cargill, Berne Ferry, Rob Aalberse, Eleanor Barnes, Theo Rispens

**Affiliations:** ∗ Translational Gastroenterology Unit, John Radcliffe Hospital, Oxford, United Kingdom; ‡ Nuffield Department of Medicine, Oxford University, Oxford, United Kingdom; § Clinical Immunology Department, Churchill Hospital, Oxford, United Kingdom; ‖ Cellular Pathology Department, Southampton General Hospital, Southampton, United Kingdom; ¶ Immunopathology Department, Sanquin, Amsterdam, The Netherlands

**Keywords:** Inflammation, Pancreas, Immune Response, Detection, AIP, autoimmune pancreatitis, CI, confidence interval, DC, disease control subjects, FcεRI, Fc Epsilon Receptor 1, HC, healthy control subjects, HPF, high power field, IgG4, immunoglobulin G subclass 4, IgG4-RD, IgG4-related disease, IL, interleukin

## Abstract

**Background & Aims:**

IgG subclass 4–related disease (IgG4-RD) is characterized by increased serum levels of IgG4 and infiltration of biliary, pancreatic, and other tissues by IgG4-positive plasma cells. We assessed the prevalence of allergy and/or atopy, serum, and tissue IgE antibodies, and blood and tissue eosinophils in patients with IgG4-RD. We investigated the association between serum IgE and diagnosis and relapse of this disease.

**Methods:**

We performed a prospective study of 48 patients with IgG4-RD, 42 patients with an increased serum level of IgG4 with other inflammatory and autoimmune conditions (disease control subjects), and 51 healthy individuals (healthy control subjects) recruited from Oxford, United Kingdom from March 2010 through March 2014, and followed for a median of 41 months (range, 3–73 months). Serum levels of immunoglobulin were measured at diagnosis, during steroid treatment, and at disease relapse for patients with IgG4-RD; levels at diagnosis were compared with baseline levels of control subjects. Allergen-specific IgEs were measured using the IgE ImmunoCAP. Levels and distribution of IgG4 and IgE antibodies in lymphoid, biliary, and pancreatic tissues from patients with IgG4-RD and disease control subjects were measured by immunohistochemistry. We analyzed data using the Spearman rank correlation and receiver operating characteristic curves.

**Results:**

Serum levels of IgG4 increased to 1.4 g/L or more, and IgE increased to 125 kIU/L or more, in 81% and 54% of patients with IgG4-RD, respectively, compared with 6% and 16% of healthy control subjects (*P* < .0001). Peripheral blood eosinophilia was detected in 38% of patients with IgG4-RD versus 9% of healthy control subjects (*P* = .004). Of patients with IgG4-RD, 63% had a history of allergy and 40% had a history of atopy with an IgE-specific response; these values were 60% and 53% in patients with increased serum levels of IgE (*P* < .05). Level of IgE at diagnosis >480 kIU/L distinguished patients with IgG4-RD from disease control subjects with 86% specificity, 36% sensitivity, and a likelihood ratio of 3.2. Level of IgE at diagnosis >380 kIU/L identified patients with disease relapse with 88% specificity, 64% sensitivity, and a likelihood ratio of 5.4. IgE-positive mast cells and eosinophilia were observed in lymphoid, biliary, and pancreatic tissue samples from 50% and 86% of patients with IgG4-RD, respectively.

**Conclusions:**

In a prospective study, we associated IgG4-RD with allergy, atopy, eosinophilia, increased serum levels of IgE, and IgE-positive mast cells in lymphoid, biliary, and pancreatic tissue. An IgE-mediated allergic response therefore seems to develop in most patients with IgG4-RD; levels of IgE might be used in diagnosis and predicting relapse.

IgG subclass 4–related disease (IgG4-RD) is a systemic fibroinflammatory condition, of which IgG4-related sclerosing cholangitis and autoimmune pancreatitis (AIP) are the biliary and pancreatic manifestations. An elevated serum IgG4 and abundance of IgG4-positive plasma cells infiltrating the involved organs is well described.^1^ Elevated serum IgE concentrations were first reported in a series of Japanese patients with AIP; retrospective studies suggest a prevalence of between 34% and 86%.^2^, ^3^ Peripheral and tissue eosinophilia are traditionally seen in the context of atopic and allergic conditions, but have also been described in AIP.^4^ Furthermore, a clinical history of allergy can be ascertained in 40% to 80% of patients with AIP.^2^, ^5^

Strong links between IgG4 and IgE are well established. IgG4 responses are often associated with IgE-mediated allergy, but these responses are distinct. Both IgG4 and IgE are induced by Th2 cytokines interleukin (IL) 4 and/or IL13^6^; however production of IgE antibodies often occurs well before IgG4 antibodies appear.^7^ It is also common to find IgG4 antibodies in the absence of IgE antibodies, a process called the “modified Th2 response.”^8^ An important regulatory component in the modified Th2 response is IL10, which promotes the switch to IgG4 and inhibits IgE production.^9^ IgG4 responses require frequent and/or high antigen exposure and are observed in situations associated with tolerance induction, such as during allergen immunotherapy. This can result in an increase of IgG4 (10–100 fold), along with a gradual decline in antiallergen IgE antibodies and symptoms of allergic rhinitis and asthma. Th2-cell-dominant immune responses are reportedly increased in the peripheral blood and tissue of IgG4-RD patients, and abundant regulatory cells producing IL10 and transforming growth factor-β infiltrate affected organs.^10^, ^11^, ^12^

In this paper, we set out to establish the prevalence of allergy and atopy, blood and tissue eosinophilia, serum total and allergen-specific IgE responses, and tissue IgE antibodies in a prospective UK cohort of IgG4-RD patients. We sought to determine the utility of serum IgE in differentiating patients with IgG4-related sclerosing cholangitis/AIP from disease mimics with an elevated serum IgG4, such as primary sclerosing cholangitis, and its relationship with corticosteroid use and disease relapse.

## Methods

### Patient and Control Recruitment

For this study, 48 IgG4-RD patients, 42 disease control subjects with an elevated serum IgG4 (DC), and 51 healthy control subjects (HC) were recruited from the John Radcliffe Hospital, Oxford, United Kingdom, between March 2010 and March 2014, and followed-up for a median of 41 months (range, 3–73 months). Diagnostic criteria for each group are defined in the Supplementary Methods.

### Serologic Testing for IgG, IgG Subclass 4, and IgE

Serum immunoglobulins were measured at diagnosis, during steroid treatment, and at disease relapse within the IgG4-RD cohort. Comparisons among IgG4-RD patients, DC, and HC were made at diagnosis before steroid initiation. Total serum IgG and IgG4 were measured by nephelometry (BNII analyser, Siemens, Oxford, UK). Total IgE was measured by the automated Immunocap method (Phadia 250, Milton Keynes, UK). Elevated serum IgG (≥16 g/L), IgG4 (≥1.4 g/L), and IgE (≥125 kIU/L) were defined by institution range.

### IgE-Specific ImmunoCAP

An IgE ImmunoCAP method was used to determine and quantify allergen-specific IgE antibodies. IgE-specific allergen testing was performed using a set of allergen mixes: grass pollen, mold, tree pollen, and nut (Phadia, Thermo Scientific, Uppsala, Sweden), as specified in the Supplementary Methods.

### Peripheral and Tissue Eosinophilia

Peripheral blood eosinophil count was measured at diagnosis. An elevated eosinophil count was defined as >500 cells/μL (count >0.5). Tissue eosinophil number was graded as none (0), occasional (<10), moderate (10–25), or marked (>25) on hematoxylin-eosin specimens by the reporting pathologist.

### Immunostaining for IgG, IgG Subclass 4, and IgE Antibodies

Tissues were assessed for morphology and immunostained with IgG and IgG4 monoclonal antibody and IgE polyclonal antibody, as described in the Supplementary Methods. Double immunostaining for IgE and CD138 (plasma cells) or mast cell tryptase (mast cells) was performed.

### Statistical Analysis

Comparisons among groups were analyzed with a 2-tailed Mann-Whitney test; correlations quantified as Spearman rank correlation coefficient. Statistics and receiver operating characteristic curve analysis were performed using Graphpad Prism (Graphpad Software, San Diego, CA) version 6.0. A *P* < .05 was considered significant.

## Results

### Prevalence of Serum IgG Subclass 4 and IgE Elevation

**Figure 1:**
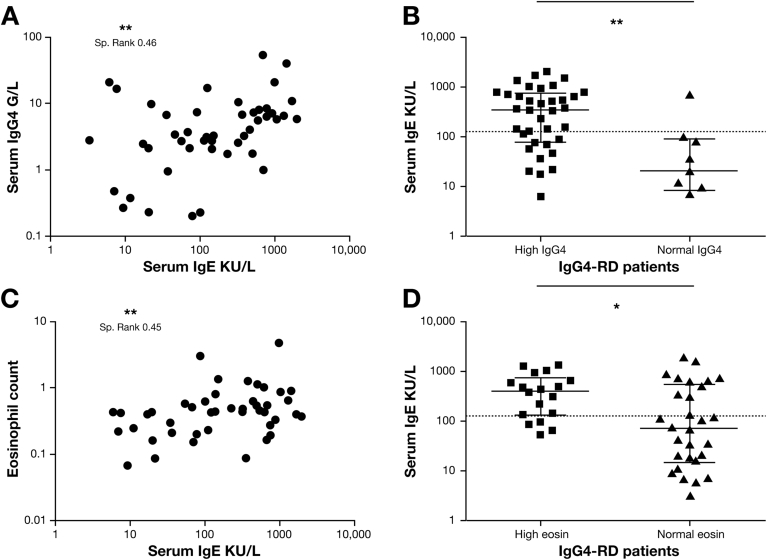
The relationship of serum IgE with serum IgG4 and peripheral blood eosinophil counts in IgG4-RD. (*A*) Correlation plots showing serum IgE (kIU/L) plotted against serum IgG4 (g/L) in IgG4-RD. (*B*) Dot plots of serum IgE in IgG4-RD patients with a high (≥1.4 g/L) or normal serum IgG4. *Dashed line* is the serum IgE upper limit of normal (≥125 kIU/L). (*C*) Correlation plots showing blood eosinophil count (cells/μL) plotted against serum IgE (kIU/L) in IgG4-RD. (*D*) Dot plots of serum IgE in IgG4-RD patients with a high (≥500 cells/μL) or normal eosinophil count. *Dashed line* is the serum IgE upper limit of normal (≥125 kIU/L). Spearman rank correlation and *P* values are expressed as NS ≥0.05, *P < .05, **P < .01. Mann-Whitney *P* values, where NS *P* ≥ .05, *P < .05.

**Supplementary Figure 1:**
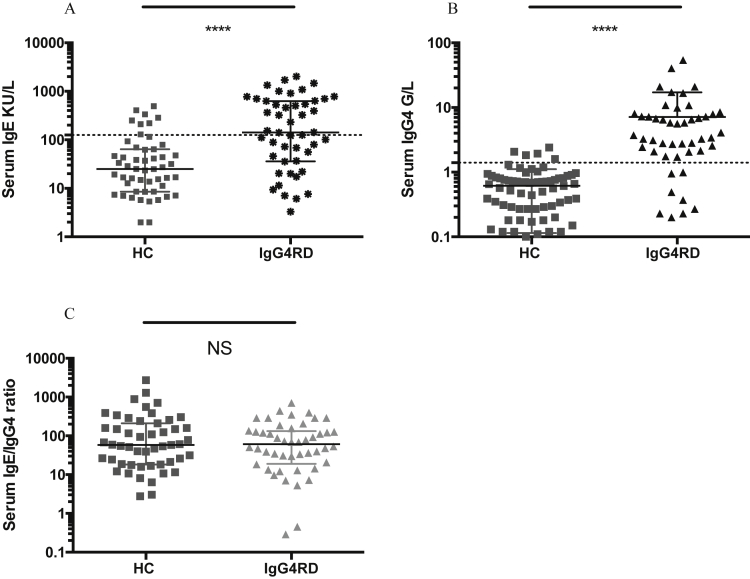
Serum IgG4 and IgE levels and IgE/IgG4 ratio in IgG4-RD and HC. Dot plots showing (*A*) serum IgG4 concentrations, (*B*) serum IgE concentrations, (*C*) IgE/IgG4 ratio (kIU/g), in IgG4-RD patients and HC. The y-axis shows the serum IgE concentration (KU/L), serum IgG4 (g/L), or IgE/IgG4 ratio. *Dashed line* is the serum IgE upper limit of normal (≥125 kIU/L) and serum IgG4 upper limit of normal (≥1.4 g/L). Mann-Whitney *P* values; NS *P* < .05, ****P < .0001.

**Supplementary Figure 2:**
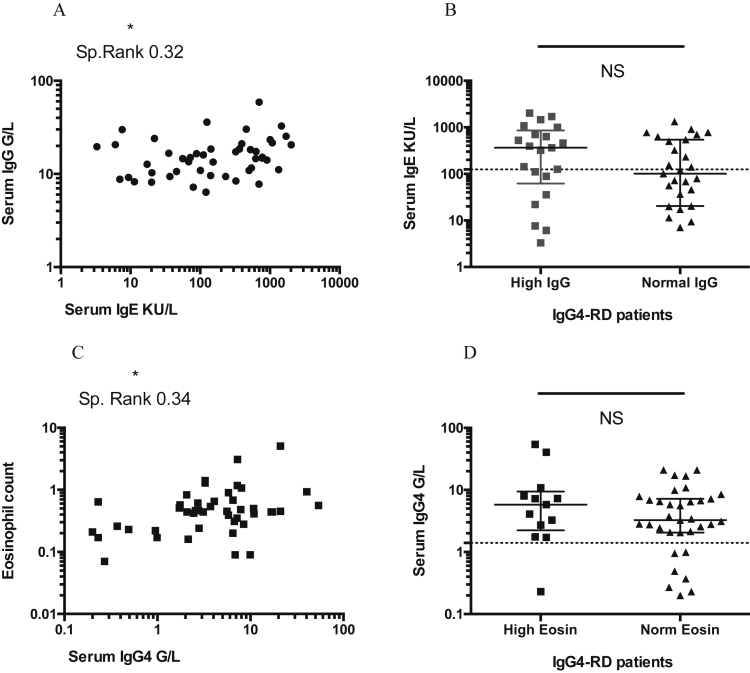
The relationship of serum IgE with serum IgG, and serum IgG4 with peripheral blood eosinophil count, in IgG4-RD. (*A*) Correlation plots showing serum IgE (kIU/L) concentration plotted against serum IgG (g/L) concentrations in IgG4-RD. (*B*) Dot plots of serum IgE concentrations in IgG4-RD patients with a high (≥16.0 g/L) or normal serum IgG. *Dashed line* is the serum IgE upper limit of normal (≥125 kIU/L). (*C*) Correlation plots showing blood eosinophil count (cells/μL) plotted against serum IgG4 (g/L) concentrations in IgG4-RD. (*D*) Dot plots of serum IgG4 concentrations in IgG4-RD patients with a high (≥500 cells/μL) or normal eosinophil count. *Dashed line* is the serum IgE upper limit of normal (≥125 kIU/L). Spearman rank correlation coefficient and *P* values are expressed as NS ≥0.05, *P < .05, Mann-Whitney *P* values, where NS *P* ≥ .05, *P < .05. Sp., Spearman.

The serum IgG, IgG1, IgG4, and IgE levels at diagnosis were higher in IgG4-RD patients than in HC (*P* < .0001), as shown in Table 1 (Supplementary Figure 1*A* and *B*). There was no difference in the IgE/IgG4 ratio between the 2 groups (Supplementary Figure 1*C*). Elevated IgG4 concentrations were demonstrated in 81% (39 of 48), and elevated IgE in 54% (26 of 48) of the cases, both more prevalent compared with HC (*P* < .0001) (Table 1). There was a positive correlation between serum IgE and IgG4 (Spearman rank, 0.46; 95% confidence interval [CI], 0.18–0.66; *P* = .001) (Figure 1*A*), and serum IgE and IgG (Spearman rank, 0.32; 95% CI, 0.028–0.56; *P* = .028) (Supplementary Figure 2*A*). However, there was no difference between serum IgE levels in IgG4-RD patients with a normal and high IgG level (*P* = .178) (Supplementary Figure 2*B*). Serum IgE levels were increased in the high IgG4 compared with normal IgG4 group (*P* = .017) (Figure 1*B*). In the 9 IgG4-RD patients with a normal serum IgG4 at diagnosis, only 1 had an elevated IgE.

**Table 1:** Clinical Characteristics and Laboratory Measurements in IgG4-RD Patients and Healthy Control Subjects

	IgG4-RD patients n = 48	Healthy control subjects n = 51	*P* values
IgG median (range), *g/L*	14.7 (6.36–59.1)	10.75 (6.33–16.0)	< .0001****
IgG1 median (range), *g/L*	8.38 (3.94–32.2)	6.65 (3.41–10.6)	< .0001****
IgG4 median (range), *g/L*	3.57 (0.0–54.1)	0.57 (0.0–2.4)	< .0001****
IgE median (range), *kIU/L*	142.0 (0.0–2024)	25.1 (1.99–491.0)	< .0001****
IgE/IgG4 ratio median (range), *kIU/g*	69.19 (0–705.1)	57.93 (0–2728)	.6491 NS
Eosinophils median (range) count	0.44 (0.0–5.05)	0.16 (0.05–0.78)	< .0001****
Clinical history of atopy/allergy (*%*)	30/48 (62.5)	6/35 (17.1)	< .0001****
High serum IgG4 >1.4 g/L (*%*)	39/48 (81.3)	3/51 (5.9)	< .0001****
High serum IgE >125 kIU/L (*%*)	26/48 (54.2)	8/51 (15.7)	.0001***
Serum eosinophilia >0.5 count (*%*)	18/48 (37.5)	3/35 (8.6)	.0042**

NOTE. *P* values were calculated by using Mann-Whitney for comparison between 2 groups and Fisher exact test for categorical variables, where NS *P* ≥ .05, **P = .01, ***P = .001, ****P < .0001.

### Prevalence of Peripheral Eosinophilia

Peripheral blood eosinophilia was present in 38% of IgG4-RD (median, 0.44; range, 0.0–5.05) versus 8.6% of HC (median, 0.16; range, 0.05–0.78) (*P* = .004) (Table 1). There was a positive correlation between eosinophil count and serum IgE (Spearman rank, 0.45; 95% CI, 0.18–0.65; *P* = .001) (Figure 1*C* and *D*) and serum IgG4 (Spearman rank, 0.34; 95% CI, 0.049–0.57; *P* = .019) (Supplementary Figure 2*C*). However, there was no difference between serum IgG4 levels in IgG4-RD patients with a normal and high eosinophil count (*P* = .298) (Supplementary Figure 2*D*).

### Prevalence of Allergy and Atopy

**Supplementary Figure 3:**
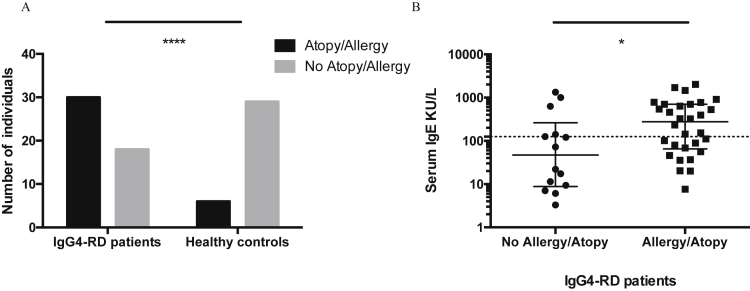
Allergy and/or atopy and serum IgE levels in IgG4-RD patients and healthy control subjects. (*A*) Bar chart showing the number of IgG4-RD patients and healthy control subjects with (*black*) or without (*grey*) a history of allergy and/or atopy. (*B*) Dot plot showing serum IgE concentration (kIU/L) in IgG4-RD patients with and without a history of allergy and/or atopy. *Dashed line* is the serum IgE upper limit of normal (≥125 kIU/L). Mann-Whitney *P* values, where *P < .05, ****P < .0001.

A history of allergy was identified in 63% of patients with IgG4-RD compared with 17% of HC (*P* < .001) (Table 1, Supplementary Figure 3*A*), and national UK statistics of 20% (http://www.allergyuk.org/allergy-statistics/allergy-statistics). There was a higher serum IgE in IgG4-RD patients with an allergic history than those without (*P* = .031) (Supplementary Figure 3*B*). An elevated serum IgE level was present in 60% of IgG4-RD patients with allergy. No patient had a history or evidence of parasitic infection.

Atopy was defined by evidence of an IgE antibody response in addition to clinical symptoms. An allergen-specific IgE response was identified in 52% (25 of 48) of IgG4-RD patients and 40% (19 of 48) of IgG4-RD patients were defined as “atopic.” An elevated serum IgE level was present in 53% of IgG4-RD patients with atopy. In total, 62% (16 of 26) of IgG4-RD with an elevated total serum IgE had a positive allergen-specific IgE response and 41% (9 of 22) with a normal IgE had a positive IgE-allergen specific response (*P* = .25).

### Allergen-Specific IgE Responses

To establish if there was a particular allergen prevalent in IgG4-RD and to investigate whether an IgE-specific response was more prevalent in those with an elevated total IgE, we tested the serum of 16 patients with elevated IgE and 17 patients with normal IgE levels to 4 broad allergen panels (grass, mold, tree, and nut mixes). Three-quarters (75%) of those with an elevated IgE (12 of 16) were positive to at least 1 of these panels, whereas 24% of those with a normal IgE (4 of 17) were positive to a single grass allergen panel only (*P* = .005) (Supplementary Table 1).

### Serum IgE to Differentiate IgG Subclass 4–Related Disease From Disease Control Subjects With an Elevated Serum IgG Subclass 4

**Figure 2:**
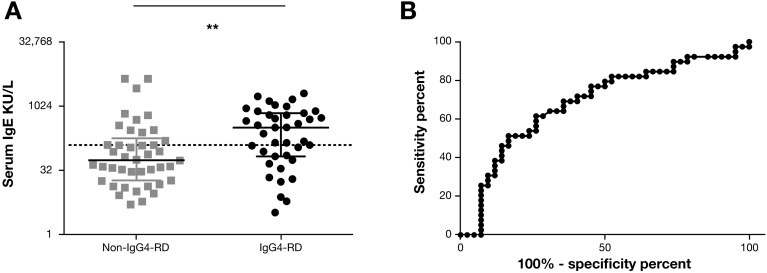
Serum IgE levels and receiver operating characteristic curve to differentiate IgG4-RD patients and non-IgG4-RD disease control subjects with an elevated serum IgG4. (*A*) Dot plot showing serum IgE in IgG4-RD patients and non-IgG4-RD disease control subjects with an elevated serum IgG4. The y-axis shows the serum IgE concentration (kIU/L). *Dashed line* is the serum IgE upper limit of normal (≥125 kIU/L). Mann-Whitney *P* values *P < .05. (*B*) Receiver operating characteristic curve shows the sensitivity and specificity of IgE in distinguishing IgG4-RD from non-IgG4-RD conditions, with an elevated serum IgG4.

Serum IgE levels were measured in IgG4-RD patients with an elevated IgG4, and DC who had other autoimmune, inflammatory, and infective diseases and an elevated IgG4, to investigate if IgE levels may help to discriminate the 2 groups. The groups were well matched in age (*P* = .21) and gender (*P* = 1.0). In those IgG4-RD patients with an elevated IgG4 (n = 39), serum IgE was higher (median, 323 kIU/L; range, 3.31–2024 kIU/L) than in the non-IgG4-RD DCs (n = 42) (median, 55.7 kIU/L; range, 5.11–4457 kIU/L) (*P* = .003) (Figure 2*A*). The ability of serum IgE to distinguish IgG4-RD from DC is shown on the receiver operating characteristic curve; area under the curve was 0.69 (*P* = .004; 95% CI, 0.57–0.81) (Figure 2*B*).

At a serum IgE cutoff of 125 kIU/L, sensitivity was 67%, specificity was 64%, negative predictive value was 68%, and positive predictive value was 63%, with a likelihood ratio of 1.8 to distinguish IgG4-RD from non-IgG4-RD DCs with an elevated IgG4. At a serum IgE of 480 kIU/L (four times the upper limit of normal), the sensitivity fell to 36%, specificity increased to 86%, positive predictive value was 70%, and negative predictive value was 59%, with a likelihood ratio of 3.2.

### Serum IgE and Corticosteroid Treatment

**Supplementary Figure 4:**
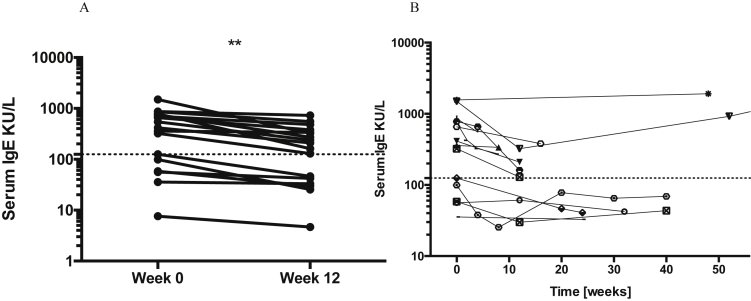
Serum IgE and relationship to corticosteroid therapy in IgG4-RD. (*A*) Serum IgE concentrations in IgG4-RD patients at diagnosis before corticosteroids (0 weeks) and after 12 weeks of corticosteroid therapy (n = 15). On the y-axis is serum IgE concentrations kIU/L. *Dashed line* is the upper limit of IgE (≥125 kIU/L). Two-tailed paired Student *t* test *P* values, where **P < .01. (*B*) Serum IgE concentrations in IgG4-RD patients during follow-up over 12 months of corticosteroid therapy. The *dashed line* is the same as in *A*.

Of 48 IgG4-RD patients, 79% (38 of 48) received corticosteroid therapy, 15% (10 of 48) underwent surgical resection, and 6.2% (3 of 48) had conservative management because of severe diabetes (1), end-stage cirrhosis (1), and patient preference (1). In those who received corticosteroid therapy with available serum samples (n = 15), IgE levels were lower compared with pretreatment levels by 12 weeks (*P* < .001) (Supplementary Figure 4*A*). Over time, serum IgE levels plateaued or increased, corresponding to corticosteroid discontinuation and clinical remission (Supplementary Figure 4*B*).

### Serum IgE and Disease Relapse

**Figure 3:**
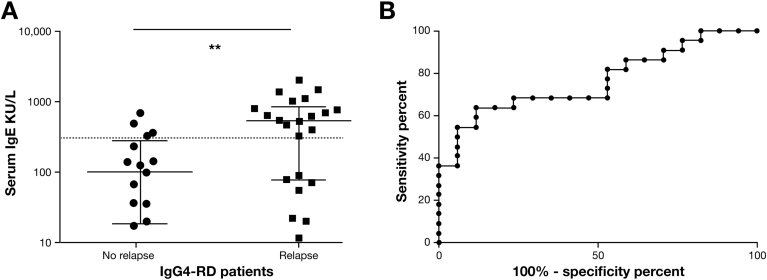
Serum IgE levels and receiver operating characteristic curve for disease relapse in IgG4-RD patients. (*A*) Dot plot showing serum IgE in IgG4-RD patients with and without evidence of biochemical and radiologic disease relapse. The y-axis shows the serum IgE concentration (kIU/L). *Dashed line* indicates a serum IgE of 380 kIU/L). Mann-Whitney *P* values NS *P* ≥ .05, **P < .01. (*B*) Receiver operating characteristic curve shows the sensitivity and specificity of IgE at diagnosis in determining disease relapse in IgG4-RD patients.

Of 48 IgG4-RD patients, 43 had sufficient follow-up (>6 months) to determine evidence of relapse. At least 1 episode of biochemical and/or radiologic relapse was recorded in 54% (23 of 43). Serum IgE at diagnosis was higher in those IgG4-RD patients who relapsed than those who did not (*P* = .001) (Figure 3*A*). Receiver operating characteristic curve analysis yielded an area under the curve of 0.76 (*P* = .005; 95% CI, 0.62–0.91). Using an IgE cutoff of 380 kIU/L, the sensitivity was 64%, specificity was 88%, with a likelihood ratio of 5.4, to predict relapse (Figure 3*B*). An IgE of >125 kIU/L at diagnosis did not predict relapse.

### Prevalence of Tissue Eosinophilia

Histologic specimens were available from 77% (37 of 48) of IgG4-RD patients for morphologic review and immunostaining; 15 resection and 32 biopsies. Tissue eosinophils were present in 86% (32 of 37) of specimens. Eosinophil infiltration was graded as “moderate” (10–25 eosinophils/high power field [HPF]) or “marked” (>25 eosinophils/HPF) in 73% (27 of 37) of specimens.

### Prevalence and Distribution of Tissue IgG Subclass 4 and IgE-Positive Cells

Tissue IgG4 immunohistochemistry was performed in all 37 IgG4-RD specimens; the IgG4-positive plasma cell count >10/HPF (range, 5–130/HPF) in 95% (35 of 37). The 2 patients with biopsy IgG4 counts <10/HPF had resection specimens with morphologic characteristics and ratios “highly suggestive” of IgG4-RD.

**Figure 4:**
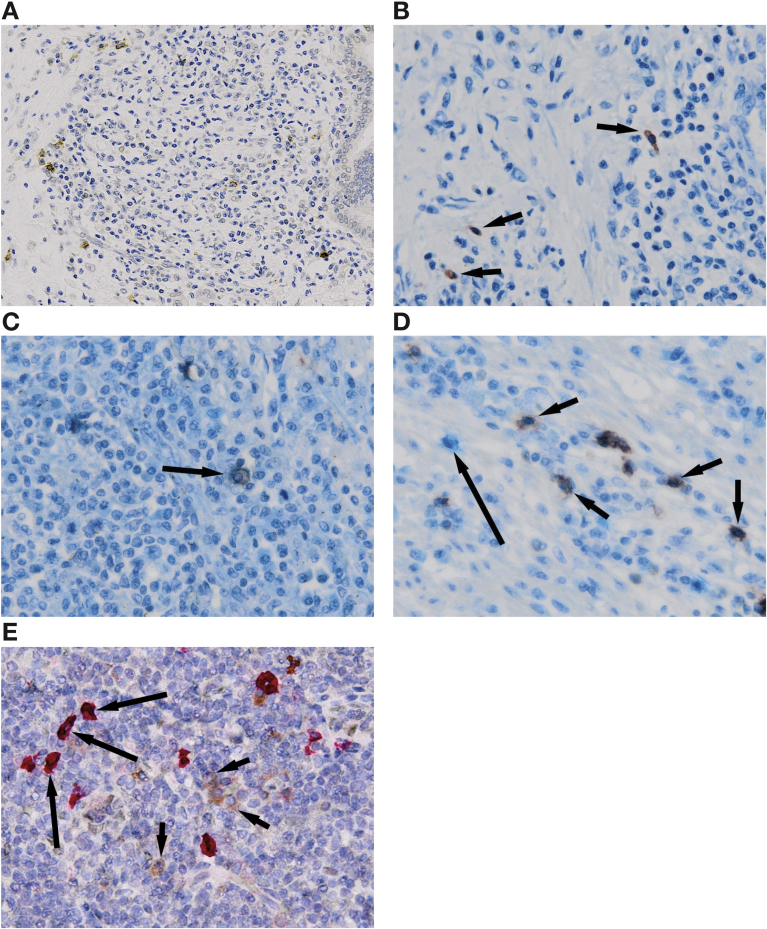
Immunohistochemical staining for inflammatory cell subsets and IgE in IgG4-RD. (*A*) Type 1 autoimmune pancreatitis, showing IgE-positive cells (*brown*) within the inflammatory cell infiltrate (IgE immunohistochemistry, original magnification ×200). (*B*) IgG4-related sialadenitis, showing mast cells (*red*; *arrows*) within the inflammatory cell infiltrate (mast cell tryptase immunohistochemistry, original magnification ×400). (*C*) Type 1 autoimmune pancreatitis, showing a mast cell (*red* cytoplasm) expressing surface IgE (*pale blue*, in contrast to *dark blue* hematoxylin counterstain; *arrow*) within the inflammatory infiltrate (mast cell tryptase [*red*] and IgE [*pale blue*] double immunohistochemistry, original magnification ×400). (*D*) Type 1 autoimmune pancreatitis, showing CD20-positive B-cells (*red*; *short arrows*) not expressing IgE; and a separate inflammatory cell (not CD20-positive) expressing IgE (*pale blue*, in contrast to *dark blue* hematoxylin counterstain; *long arrow*) within the inflammatory cell infiltrate (CD20 [*red*] and IgE [*pale blue*] double immunohistochemistry, original magnification ×400). (*E*) Nasal polyp, showing CD138-positive plasma cells (*bright red*; *long arrows*) and a separate population of CD138-negative and IgE-positive cells (*brown*; *short arrows*) within the inflammatory cell infiltrate (CD138 [*bright red*] and IgE [*brown*] double immunohistochemistry, original magnification ×400).

Tissue IgE immunohistochemistry was performed in 8 IgG4-RD and 6 DC specimens (Supplementary Table 2). The IgE-positive cell count in IgG4-RD (median, 10 cells/HPF; range, 1–30 cells/HPF) was higher than DC (median, 4 cells/HPF; range, 0**–**10 cells/HPF) (*P* = .15) tissues. The IgE-positive cell count was >10/HPF in 50% of samples stained (4 of 8). In IgG4-RD tissues, IgE-positive cells were localized to the mantle zone of the germinal center, within lymphoid aggregates and also scattered throughout the inflammatory infiltrate (Figure 4*A*). The IgE-positive cell staining was both nuclear and surface. Mast cells were also scattered throughout the inflammatory infiltrate (Figure 4*B*). IgE-positive cells and mast cells had a similar distribution, and costaining demonstrated evidence of IgE-positive mast cells (mast cell cytoplasmic staining and IgE peripheral staining) in IgG4-RD tissue (Figure 4*C*). IgE-positive cells stained separately to CD20-positive B cells (Figure 4*D*) and CD138-positive plasma cells (Figure 4E). This suggests IgE-positive cells represent IgE attachment to mast cells, although we cannot exclude the possibility that a proportion could be IgE-producing B cells.

In 3 IgG4-RD patients with the highest mean count of IgE-positive mast cells, a retrospective serum mast cell tryptase was performed (presteroid samples), which were within the normal range.

### Red Flags for the Diagnosis of IgG Subclass 4–Related Disease

**Figure 5:**
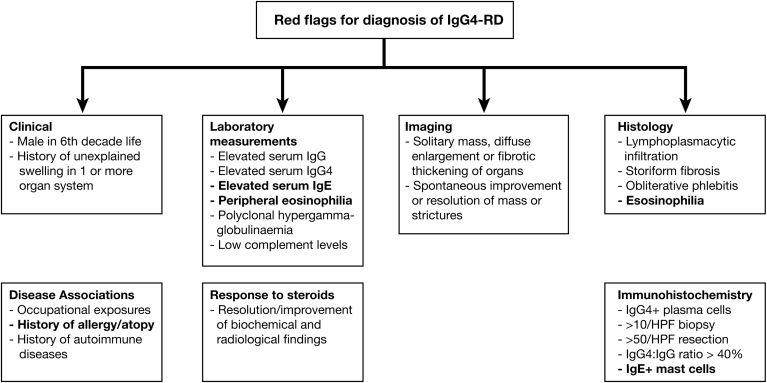
Red flags in the diagnosis of IgG4-RD. A set of red flags to raise suspicion of a diagnosis of IgG4-RD.

Red flags, incorporating these findings, are shown in Figure 5.

## Discussion

In the original landmark study of elevated serum IgG4 in patients with AIP in Japan, there was no difference reported in IgE levels between AIP and HC.^13^ Subsequent retrospective studies reported an elevated IgE in 34%–86% of patients with AIP.^2^, ^3^ In our prospective study of IgG4-RD, an elevated IgE was found in 57% at diagnosis, and a positive correlation was shown between IgE and both IgG4 and eosinophils count. We report a frequent clinical history of allergy (63%) and atopy (40%) in IgG4-RD, supported by retrospective data in Japanese patients with AIP,^2^ but not by other groups.^3^, ^14^, ^15^ Peripheral eosinophilia and elevated IgE have also been described in subsets of IgG4-RD patients without atopy.^15^ The main discrepancy lies in the definition of atopy; symptoms plus an IgE-specific protein response, which has not been characterized in other studies. We also show a higher IgE level in those individuals with allergy/atopy, supported by studies showing IgG4-RD patients with atopy express up-regulated Th2 cytokines in peripheral blood.^15^, ^16^ We have also reported polyclonal elevations of IgG4 to multiple food allergens in IgG4-RD patients.^17^

Our results support the utility of serum IgE in diagnosis of IgG4-RD, with a level of >480 KU/L differentiating IgG4-RD from DC with an elevated serum IgG4 (specificity 86%, sensitivity 36%). This 2-dimensional diagnostic approach (ie, IgE combined with IgG4) was supported by the observation that patients with a normal serum IgG4 did not have an elevated IgE. There were no DCs with malignancy in this dataset; however, serum IgE levels may be used to distinguish organ-specific IgG4-RD from malignant lesions, potentially minimizing unnecessary surgical intervention. This approach requires further validation. An elevated IgE at diagnosis >380 KU/L was also a marker of disease relapse (specificity 88%, sensitivity 64%), defining a subgroup requiring careful follow-up. This is supported by findings in a recent retrospective cohort study of predictors of disease relapse in IgG4-RD after rituximab therapy; in 21 (37%) patients experiencing disease relapse, baseline serum IgG4, IgE, and circulating eosinophils predicted relapse.^18^

Interestingly, IgE levels decreased during the first 12 weeks of corticosteroid therapy, with a parallel fall in disease activity (and IgG4 levels^19^). This finding is in line with observations in a retrospective cohort of AIP.^5^ Furthermore, the anti-CD20 B cell depletion therapy rituximab, used in refractory disease, results in a decrease in serum IgG4 and IgE with regression of active disease.^20^ However, IgE levels do not fall with steroid treatment in atopic dermatitis^21^ and do not reflect disease activity in asthma or allergic rhinitis.^22^ This complex interplay between allergic disease and IgE levels indicates that a correlation between IgE and response to treatment does not necessarily point to a pathogenic role for IgE in IgG4-RD.

The presence of tissue IgE-positive mast cells in affected organs represents a novel finding, and suggests a possible role of an IgE-mediated response. Mast cells are involved in a variety of immune responses including chronic inflammation and autoimmune disease.^23^ IgE is a key stimulator of mast cells via binding to the high-affinity IgE receptor (FcεRI). Mast cells secrete mediators including Th2 and regulatory cytokines in response to allergens binding to specific cell-bound IgE. Nonspecific polyclonal IgE can also induce cytokine secretion, independent of antigen.^24^ Furthermore, chronic elevation of IgE induces upregulation of FcεRI on mast cells, resulting in inhibition of mast cell apoptosis and promotion of cytokine production.^25^ Indeed, this may explain the predominantly surface IgE-positive mast cell staining seen in our tissue specimens, caused by high levels of membrane-associated IgE. Mast cells might also contribute to fibrosis,^26^ supported by our observation of IgE-positive mast cell infiltration in an IgG4-related fibrosclerotic mesenteric mass (Supplementary Table 2).

The etiology of the elevated IgG4 and IgE in the blood and tissue of IgG4-RD patients remains unexplained. Both IgG4 and IgE require Th2 cytokines, IL4 and IL13, for induction. By contrast, IL10 promotes IgG4-switched cells, but downregulates IgE.^9^ Although we have shown that tissue IgE-positive cells represent IgE attachment to mast cells, a proportion could also be IgE-producing B cells. IgE-producing memory B cells and plasma cells may arise directly through a germinal center IgE-intermediate cell^27^, ^28^ but also indirectly from class switching of IgG4 or IgG1 B cells.^29^ Both situations could lead to elevated IgE and IgG4 in IgG4-RD, the former by sharing of Th2 conditions and the latter in a higher frequency switch from IgG4 cells. Of note, IgG4 B cells seem to have increased capacity to capture IgE molecules, by upregulation of the FcεRII on their surface,^30^ suggesting a functional link between IgE and IgG4 B cell responses. Importantly, dysfunction of immune cells, such as Th2 and T regulatory cells, and cytokines IL4, IL13, and IL10, may pave the way for increasing serum IgE levels and IgE-positive cells in the tissues of patients with IgG4-RD.

The discovery of IgE, eosinophilia, and mast cell infiltration in IgG4-RD highlights novel therapeutic options. The prostaglandin D_2_ receptor (CRTh2), important in allergic inflammation, is expressed on Th2 cells; innate cells, such as eosinophils; and responds to mast cell–derived factors.^31^, ^32^ The frequency of CRTh2 cells was increased in IgG4-siloadenitis, with a nonsignificant correlation with IgE and eosinophils.^33^ We also report upregulation of PGD2 and CRTh2 in gene expression analysis of patients with IgG4-related sclerosing cholangitis/AIP.^34^ Blockade of CRTh2 can reduce allergic inflammation in rodent models of antigen-induced airway inflammation, allergic rhinitis, and atopic dermatitis, and may be effective in a subgroup of IgG4-RD patients with atopy.

In summary, this prospective study provides new evidence for the relationship of serum and tissue IgE antibodies in patients with IgG4-RD. Firstly, it supports the measurement of serum IgE levels at diagnosis, to help differentiate IgG4-RD from non-IgG4-RD conditions, and to determine the risk of subsequent disease relapse in those with IgG4-RD. Secondly, it implicates an IgE-mediated allergic response in a subgroup of patients, supported by a history of allergy and atopy, peripheral and tissue eosinophilia and the presence of IgE+ mast cells in affected tissues, which enables novel therapeutic avenues to be explored.

## Figures and Tables

**Supplementary Table 1:** Serum IgE and Allergen-Specific IgE Responses to 4 Allergen Panels

Serum IgE	Number (*%*) of patients				
	Grass mix	Mold mix	Tree mix	Nut mix	Total
Elevated IgE n = 16	8/16 (50)	2/16 (12.5)	3/16 (18.8)	7/16 (43.8)	12/16 (75)
Normal IgE n = 17	4/17 (23.5)	0/17 (0)	0/17 (0)	0/17 (0)	4/17 (23.5)

NOTE. Number and percentage of positive responses to allergen-specific IgE from 4 different allergen panel mixes, each panel containing 8 individual allergens. The groups are divided into patients with a normal and elevated IgE (>125 kIU/L) concentration.

**Supplementary Table 2:** IgE Immunostaining in IgG4-RD Patients and DC

Disease	Sample type	Specimen type	Mean of 3 IgE-positive cells/HPF	Description of IgE-positive cell staining
IgG4-RD	Mesenteric mass in IgG4-FS	R	7	Scattered IgE-positive cells, diffuse peripheral and central GC staining, cells in areas of fibrosis
	Whipples in AIP	R	18	Numerous scattered IgE-positive cells with nuclear staining
	CBD in AIP and IgG4-SC	R	7	Scattered IgE-positive cells only
	Submandibular gland in IgG4-SA	R	12	Scattered IgE-positive cells and diffuse GC staining
	Whipples in AIP	R	19	Cell surface and nuclear staining; PC in close proximity
	Whipples in AIP	R	1	Scattered IgE-positive cells only
	Pancreas and CBD in AIP	R	16	Scattered diffuse IgE-positive cells, ductal inflammation, duodenum many IgE-positive cells
	Liver in IgG4-SC	B	2	Scattered IgE-positive cells around portal tracts
Disease controls	Reactive lymph node	R	1	Paracortex and medulla staining
	Gallbladder in chronic cholecystitis	R	3	Scattered IgE-positive cells only
	Pancreas in chronic pancreatitis	R	10	IgE-positive cells accumulate in lymphoid aggregates
	CBD and liver specimen in secondary sclerosing cholangitis	R	8	IgE-positive cells accumulate in lymphoid aggregates, and in periductal fibrosis
	Pancreas in chronic pancreatitis	R	0	No IgE-positive cells
	Nasal polyp	B	5	IgE-positive cells accumulate in lymphoid aggregates and in GC

NOTE. The specimen, mean IgE count, and cell distribution in tissue sections from IgG4-RD patients (n = 8) and disease controls (n = 6).

B, biopsy; CBD, common bile duct; GC, germinal centers; IgG4-FS, IgG4-mesenteric fibrosclerosis; IgG4-SA, IgG4-related sialadenitis; PC, plasma cells; R, resection.

## Supplementary Methods

### Diagnostic Criteria

The diagnosis of AIP and IgG4-SC was made in accordance with the Mayo HISORt criteria^1^ and the International Consensus Diagnostic Criteria.^2^ Patients with type II AIP were excluded.^3^ Patients with extrapancreatic disease were diagnosed using the Japanese Comprehensive Diagnostic Criteria for systemic IgG4-RD.^4^ The Boston Consensus Histopathological Criteria for IgG4-RD were applied to all patients with biopsy and resection specimens available.^5^ Most patients in the cohort had AIP and/or IgG4-SC (85%) with extrapancreatic manifestations in 72% of patients.

DCs had an elevated level of serum IgG4 but had no other evidence to support a diagnosis of IgG4-RD. These included 25 patients with primary sclerosing cholangitis,^6^ 4 patients with hepatitis (1 autoimmune, 2 viral, 1 alcoholic), 3 patients with chronic cholecystitis, 4 patients with cirrhosis (3 alcoholic, 1 cryptogenic), 1 patient with sarcoidosis, 1 patient with hypereosinophilic syndrome, 1 patient with coeliac disease, 1 patient with chronic pancreatitis, 1 patient with recurrent pneumonia, and 1 patient with a pleural effusion. Healthy donors had no known immune or inflammatory disease, and were gender-matched to IgG4-RD patients and DC. All comparisons between DC and IgG4-RD patients were made at the time of diagnosis of IgG4-RD (presteroids or immunosuppressive therapy). Only DC that had not had steroid or immunosuppressive therapy at recruitment were included.

### Definitions of Allergy and Atopy

Allergy and atopy were defined in accordance with the European Academy of Allergy and Clinical Immunology classification. Allergy is a hypersensitivity reaction mediated by immunologic mechanisms (antibody- or cell-mediated). Atopy is a personal or family tendency to produce IgE antibodies in response to low-dose allergen, and as a consequence develop typical symptoms.

### Serologic Testing for IgG, IgG4, and IgE

For patients with multiple immunoglobulin measurements, the first IgG4 level recorded was used. The prozone effect (falsely low IgG4 values) was accounted for using serial dilutions where necessary.^7^ For IgE, units are expressed as kIU/L, equivalent to 1 IU/mL, and equivalent to 2.4 ng/mL.

### IgE-Specific ImmunoCAP Allergen Mixes

The allergen mixes contained the following: grass mix (GX1) of timothy grass, rye grass, meadow fescue, and meadow grass (Kentucky Blue); tree mix (TX1) of birch, box elder, oak, cedar elm, and walnut; nut mix (FX1) of peanut, coconut, hazelnut, Brazil nut, and almond; mold mix (MX1) of *Penicillium chrysogenum*, *Cladosporium herbarum*, *Aspergillus fumigatus*, and *Alternaria alternata*.

If a particular allergen was highlighted from the clinical history additional allergen testing was performed to confirm this, including house dust mite, cat dander, wheat, milk, and latex.

### Tissue Eosinophilia

Tissue eosinophil number was graded as none (0), occasional (<10), moderate (10–25), or marked (>25) on hematoxylin-eosin specimens by the reporting pathologist (A.C.B.). The number of eosinophils considered “physiologically normal” in a tissue sample depends on the organ sampled and the size of HPF used. There are currently no guidelines to define these cutoffs, except in the context of eosinophilic esophagitis where >15 eosinophils per HPF in esophageal squamous mucosa is considered diagnostic with a compatible clinical situation.^8^

### Tissue Morphology

All biopsy and resection specimens were assessed for classical morphologic features of IgG4-RD, specifically a lymphoplasmacytic infiltrate, storiform fibrosis, and obliterative phlebitis in accordance with the Boston histologic criteria.^5^ The background inflammatory infiltrate was determined using CD4 and CD8 (T cells), CD20 (B cells), CD68 (macrophages), and CD138 (plasma cells) antibodies.

### Tissue IgG4 Immunostaining

Tissues were immunostained with IgG and IgG4 monoclonal antibody in the Histopathology Department at the John Radcliffe Hospital. The IgG4 count was reported as the average number of IgG4-positive plasma cells in 3 HPF. An elevated IgG4 count in a biopsy specimen was defined as >10/HPF and in a resection specimen as >50/HPF in the pancreas or bile duct. In those with an elevated IgG4 count, an IgG4 to total IgG ratio was calculated; an elevated IgG4/IgG ratio was defined as >40% in accordance with the Boston histologic criteria.^5^

### Tissue IgE Immunostaining

Eight IgG4-RD specimens (7 resection, 1 biopsy) and 6 DC specimens (5 resection, 1 biopsy) were immunostained for IgE (Supplementary Table 1). An elevated IgE count was defined arbitrarily as >10/HPF by the reporting pathologist (A.C.B.), using the diagnostic cutoff for IgG4-positive cells in biopsy specimens in IgG4-RD. Further characterization and double immunostaining for IgE and plasma cells (CD138) or IgE and mast cells (mast cell tryptase) was performed at the Histopathology Department at Southampton General Hospital, to further define IgE-positive cells.

### Tissue Staining for B Cells, T Cells, Macrophages, and Plasma Cells

Five IgG4-RD and 5 DC biopsy specimens were stained for CD4 and CD8 (T cells), CD20 (B cells), CD68 (macrophages), and CD138 (plasma cells) to determine the background inflammatory infiltrate. In brief, 4-μm paraffin sections were cut from selected blocks and mounted onto coated slides (Thermofisher Scientific), which were dried for 20 minutes at 60°C. Slides for immunohistochemistry were prepared using the automated Prepstain Detection-system (Dako-Cytomation, Hamburg, Germany). Staining used the Dako Medical Systems System with peroxidase and diaminobenzidine solution containing 0.05% hydrogen peroxide for visualization. Interpretation of cellular staining was performed by 2 histopathologists, using a multiheaded microscope, to reduce interobserver variation.
